# Independent Predictors Associated with Patient Refusal of Invasive Diagnostic Procedures After Positive LDCT Lung Cancer Screening

**DOI:** 10.3390/diagnostics16050709

**Published:** 2026-02-27

**Authors:** Bojan Zaric, Jelena Djekic Malbasa, Tomi Kovacevic, Petar Simurdic, Vladimir Stojšić, Goran Stojanovic

**Affiliations:** 1 Faculty of Medicine, University of Novi Sad, 21000 Novi Sad, Serbia; jelena.djekic-malbasa@mf.uns.ac.rs (J.D.M.); tomi.kovacevic@mf.uns.ac.rs (T.K.); petar.simurdic@institut.rs (P.S.); vladimir.stojsic@mf.uns.ac.rs (V.S.); 2 Institute for Pulmonary Diseases of Vojvodina, 21000 Sremska Kamenica, Serbia; goran.stojanovic@institut.rs; 3 Faculty of Pharmacy Novi Sad, University Business Academy, 21000 Novi Sad, Serbia

**Keywords:** bronchoscopy, diagnostics, low dose computed tomography, lung cancer, screening

## Abstract

**Background**: Low-dose computed tomography (LDCT) screening reduces lung cancer mortality; however, the effectiveness of screening programs depends not only on detection, but also on completion of downstream diagnostic pathways following a positive screening result. Refusal of recommended invasive diagnostic procedures represents a critical but understudied form of post-screening attrition. **Methods**: This retrospective observational study was conducted within an organized multicenter LDCT lung cancer screening program in Vojvodina, Serbia. Consecutive participants screened between September 2020 and October 2025 were included. Positive screening was defined as Lung-RADS 4A, 4B, or 4X. Refusal was defined as the absence of any invasive diagnostic procedure within six months following multidisciplinary team recommendation. Demographic, clinical, smoking-related, and perceptual factors were analyzed. Time to invasive diagnostic procedures was assessed for bronchoscopy and surgical treatment. Multivariable logistic regression was used to identify factors independently associated with refusal. **Results**: Among 10,261 screened individuals, 479 (4.7%) had positive LDCT findings. Of these, 60 participants (12.5%) refused invasive diagnostic evaluation. In multivariable analysis, multimorbidity (OR 3.45, 95% CI 1.61–7.38), previous malignancy (OR 2.92, 95% CI 1.16–7.35), higher cumulative smoking exposure (OR 1.02 per pack-year, 95% CI 1.00–1.03), and screening center (Subotica vs. Novi Sad: OR 2.40, 95% CI 1.21–4.78) were independently associated with refusal of invasive diagnostic procedures. Greater concern about personal lung cancer risk was associated with a lower likelihood of refusal (OR 0.54, 95% CI 0.29–0.99). Time to bronchoscopy differed significantly across screening centers and screening years, whereas time to surgical treatment was comparable across centers and years. **Conclusions**: Refusal of invasive diagnostic procedures following positive LDCT screening represents a meaningful implementation challenge influenced by both patient vulnerability and system-level factors. Addressing modifiable barriers through improved risk communication and optimization of post-screening diagnostic pathways may enhance diagnostic continuity and strengthen the real-world effectiveness of lung cancer screening programs.

## 1. Introduction

Lung cancer remains a leading cause of cancer-related mortality worldwide, largely because the disease is frequently diagnosed at an advanced stage, when curative treatment options are limited [1]. Low-dose computed tomography (LDCT) screening in high-risk individuals has been shown to reduce lung cancer mortality by enabling earlier detection and treatment [2,3]. However, the real-world effectiveness of LDCT screening programs depends not only on imaging-based detection, but also on timely and complete downstream diagnostic pathways following a positive screening result [4].

Organized lung cancer screening with low-dose CT remains limited across much of the Balkan region, with Croatia having implemented a national program and Serbia initiating a structured organized screening initiative, while other countries currently lack formal nationwide LDCT screening programs and largely rely on opportunistic clinical practice [5].

A positive LDCT finding does not necessarily indicate malignancy, as pulmonary nodules are common in high-risk populations [6]. To balance early cancer detection against unnecessary procedures, standardized management algorithms such as Lung-RADS guide post-screening clinical decision-making [7]. Nevertheless, abnormal screening results, particularly higher-risk findings, often prompt multidisciplinary evaluation and further diagnostic work-up, including invasive procedures aimed at obtaining tissue confirmation. These procedures are essential for definitive diagnosis and treatment planning, but they also carry procedural risks, logistical demands, and challenges for healthcare system capacity [8].

In real-world screening programs, a substantial proportion of individuals with abnormal LDCT findings do not complete recommended diagnostic evaluation [9,10]. Most prior studies describe this phenomenon broadly as loss to follow-up, without clearly distinguishing between delayed evaluation, system-related barriers, and explicit refusal of invasive diagnostic procedures [11]. This lack of granularity limits understanding of where and why post-screening pathways fail in routine practice and constrains the development of targeted implementation strategies [12].

Refusal of invasive diagnostic procedures represents a distinct and clinically relevant post-screening outcome, as it occurs after individuals have already engaged with screening and received a recommendation for further evaluation [13]. Such refusal may be influenced by non-medical factors, including risk perception, prior healthcare experiences, communication quality, and organizational or geographic barriers to care [14]. Importantly, refusal should not be viewed merely as an individual decision, but rather as the result of interactions between patients and healthcare systems [15].

Understanding refusal of invasive diagnostics is particularly relevant in centralized screening models, where invasive diagnostic procedures are frequently performed at tertiary referral centers [16]. In such settings, travel distance, coordination complexity, and system organization may affect diagnostic continuity and ultimately influence the overall effectiveness of screening programs [17,18].

The aim of this study was to characterize post-screening diagnostic pathways following positive LDCT findings within an organized lung cancer screening program, with a specific focus on refusal of recommended invasive diagnostic procedures. We further aimed to identify patient-related and system-related factors associated with refusal, providing real-world evidence to inform optimization of LDCT screening implementation.

## 2. Materials and Methods

### 2.1. Study Design and Setting

This retrospective observational study was conducted within an organized low-dose computed tomography (LDCT) (GE, Revolution EVO, Milwaukee, WI, USA) lung cancer screening program in the Autonomous Province of Vojvodina, Serbia. The screening program was initiated on 20 September 2020 at the Institute for Pulmonary Diseases of Vojvodina (Novi Sad) and was subsequently expanded to additional screening sites in Subotica and Vrbas. All screening sites operated under a unified protocol for eligibility assessment, LDCT acquisition, structured reporting, and referral for further diagnostic evaluation.

The present analysis included consecutive participants screened between 20 September 2020 and 1 October 2025. Screening outcomes and downstream diagnostic events were assessed according to standardized post-screening diagnostic pathways routinely applied within the program.

### 2.2. Participants and Eligibility Criteria

Participants were eligible for inclusion in the LDCT screening program if they were aged 50–74 years at the time of screening, had no clinical signs or symptoms suggestive of lung cancer, and met predefined smoking exposure criteria. Eligible participants included current smokers with a cumulative smoking exposure of ≥30 pack-years, or ≥20 pack-years in the presence of additional lung cancer risk factors, such as chronic respiratory diseases (chronic obstructive pulmonary diseases (COPD), sarcoidosis), previous pneumonia (three or more confirmed infections in a one year period), prior malignancy other than lung cancer, a family history of lung cancer, or occupational exposure to carcinogens. Former smokers who had quit smoking within the preceding 10 years were also eligible, provided they met the same cumulative smoking exposure thresholds.

Participants were excluded from the screening program and from the present analysis if they had undergone chest computed tomography within the previous 12 months, had a prior diagnosis of lung cancer, were receiving long-term oxygen therapy, or had advanced comorbid conditions limiting life expectancy or the potential benefit of screening. Such conditions included advanced liver disease, severe COPD with hypoventilation, or New York Heart Association class IV heart failure. Severe COPD was considered an exclusion criterion only when accompanied by hypoventilation (hypercapnia) and/or long-term oxygen therapy, which would disable invasive diagnostics and treatment.

### 2.3. LDCT Acquisition and Image Interpretation

LDCT examinations were performed using site-specific multidetector CT scanners, following harmonized low-dose acquisition protocols designed to minimize radiation exposure while preserving diagnostic image quality. All LDCT scans were interpreted by radiologists experienced in thoracic imaging.

Structured reporting was applied using Lung-RADS version 2021, with standardized categorization and corresponding management recommendations assigned to each screening examination [7].

### 2.4. Definition of Screening Outcomes

A positive screening result was defined as Lung-RADS category 4A, 4B, or 4X, in accordance with Lung-RADS version 2021 recommendations. Lung-RADS category 3 was considered a finding requiring short-term imaging follow-up but was not classified as a positive screening result for the purposes of this analysis.

### 2.5. Post-Screening Diagnostic Pathway

Participants with a positive LDCT screening result entered a structured post-screening diagnostic pathway coordinated by a multidisciplinary team (MDT). Further evaluation could include follow-up imaging, positron emission tomography/computed tomography (PET/CT) when clinically indicated, and invasive diagnostic procedures aimed at obtaining tissue or cytological confirmation. The diagnostic procedures were not protocol-mandated but were performed according to routine clinical practice.

Diagnostic strategies were individualized based on lesion characteristics, anatomical accessibility, and clinical considerations. In general, bronchoscopy procedures were considered the first-line invasive diagnostic approach for lesions accessible via the bronchial tree, while transthoracic needle biopsy or surgical diagnostic procedures were employed when required related to lesion location or diagnostic feasibility. All diagnostic decisions were made within an MDT framework. Following a positive LDCT finding, diagnostic work-up was typically carried out within the same tertiary care pathway used for patients with incidentally detected lung cancer, rather than through a separate screening-specific diagnostic track.

### 2.6. Invasive Diagnostic Procedures and Definition of Refusal

Invasive diagnostic work-up was defined as any procedure performed with the intent of obtaining histological or cytological sample for pathological confirmation. These procedures included bronchoscopic techniques (with or without endobronchial ultrasound guidance), transthoracic needle aspiration or biopsy (TTNA), and surgical diagnostic procedures.

Refusal of invasive diagnostic procedures was defined as an explicit decision by a participant to decline a recommended invasive diagnostic intervention following MDT referral. Such decisions were documented in the medical records and/or screening pathway documentation; however, detailed reasons for refusal were not systematically recorded. Operationally, refusal was defined as the absence of any invasive diagnostic procedure within six months following the initial MDT recommendation. Cases in which invasive procedures were postponed or deferred due to medical contraindications, ongoing clinical evaluation, or other physician-driven reasons were not classified as refusal.

### 2.7. Data Sources and Variables

Data were collected from LDCT screening registries, radiology reports, and institutional clinical records. Extracted variables included demographic characteristics (age and sex); sociodemographic indicators (education level, marital status, employment status, and place of residence categorized as rural or urban); screening site location; and smoking-related variables (current smoking status and cumulative smoking exposure expressed in pack-years).

Clinical variables included body mass index (BMI), comorbidities, multimorbidity status, history of prior malignancy, and self-reported concern regarding personal lung cancer risk. Sociodemographic characteristics were included to explore potential social and contextual influences on post-screening diagnostic decision-making. Geographic accessibility was approximated using the screening site as a proxy indicator of travel burden to the tertiary diagnostic center. Procedure-related complications and mortality were identified through review of institutional clinical records during post-procedural follow-up.

### 2.8. Study Outcomes

The primary study outcomes were the positive screening rate, the invasive diagnostic rate among participants with positive LDCT findings, and the refusal rate of recommended invasive diagnostic procedures among LDCT-positive participants. Secondary outcomes included timing of invasive diagnostic procedures, histopathological diagnoses, procedure-related complications, and mortality.

For time-to-event analyses, invasive diagnostic or therapeutic procedures occurring more than 300 days after the index LDCT examination were excluded, as such delays were considered unlikely to reflect the screening-related diagnostic pathway.

### 2.9. Statistical Analysis

Descriptive statistics were used to summarize participant characteristics and screening outcomes. Categorical variables were presented as counts and percentages, while continuous variables were summarized as mean ± standard deviation or median with interquartile range, as appropriate.

Time-to-procedure analyses for bronchoscopy and surgical interventions were performed among participants with positive LDCT findings. Comparisons of time intervals across screening centers and screening years were conducted using the Kruskal–Wallis test. Time-to-event analyses were restricted to procedures occurring within 300 days following the index LDCT examination.

Group comparisons were performed using the χ^2^ test or Fisher’s exact test for categorical variables and appropriate parametric or non-parametric tests for continuous variables. Factors associated with refusal of invasive diagnostic procedures were explored using multivariable logistic regression analysis, with results presented as odds ratios (ORs) and 95% confidence intervals. Model calibration was assessed using the Hosmer–Lemeshow test, and discrimination was evaluated using the area under the receiver operating characteristic curve (AUC). A sensitivity analysis using a reduced model excluding the risk perception variable was performed to assess robustness of estimates. A two-sided *p*-value < 0.05 was considered statistically significant.

All statistical analyses were performed using IBM SPSS Statistics (Version 19.0, IBM Corp., Armonk, NY, USA).

### 2.10. Ethics Approval

The study was conducted in accordance with the Declaration of Helsinki and approved by the Ethics Committee of the Institute for Pulmonary Diseases of Vojvodina, Sremska Kamenica, Serbia (approval number: No.110-V/1, 22 September 2020). Written informed consent for participation in the LDCT screening program and for the use of anonymized data for research purposes was obtained from all participants.

## 3. Results

### 3.1. Study Population

**Figure 1:**
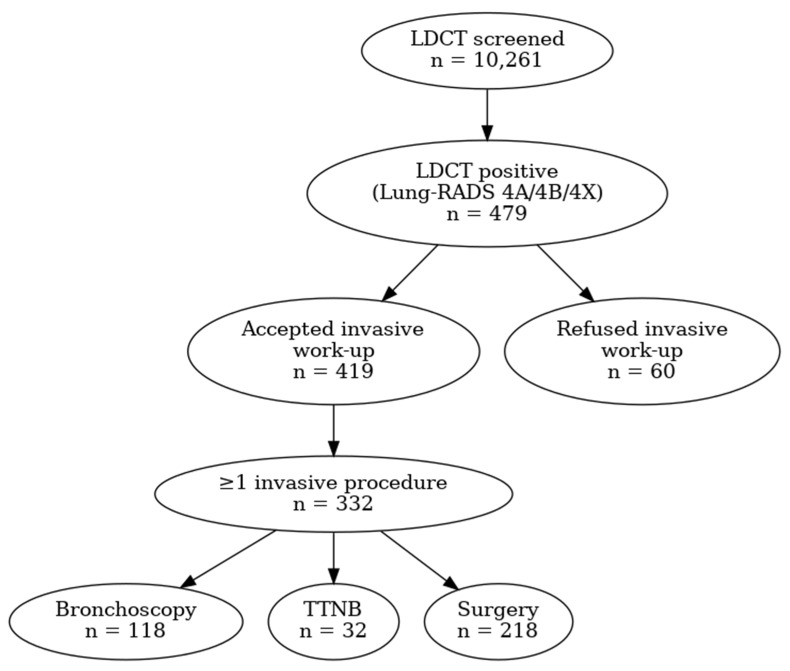
Flow diagram of participants through the LDCT screening program and post-screening diagnostic pathway.

Between 1 October 2020 and 1 October 2025, a total of 10,261 individuals underwent low-dose computed tomography (LDCT) lung cancer screening at three screening sites (Novi Sad, Subotica, and Vrbas). Among the screened population, 479 individuals (4.7%) had positive LDCT findings classified as Lung-RADS 4A, 4B, or 4X and were included in the present analysis. Among participants who accepted invasive diagnostic work-up, a proportion did not ultimately undergo an invasive procedure, reflecting alternative clinical management pathways (e.g., PET/CT surveillance) or non–patient-driven factors. These included optimization of comorbid conditions, preoperative requirements (such as temporary smoking cessation), or short-term postponement due to intercurrent illness (e.g., influenza or COVID-19). This group was analytically distinct from individuals who actively refused invasive diagnostics and was therefore not included in refusal modeling. When expressed relative to all screened participants, refusal of recommended invasive diagnostic procedures was infrequent, corresponding to 0.45% (21/4656) in Novi Sad, 0.91% (30/3286) in Subotica, and 0.39% (9/2319) in Vrbas; overall 0.58% (60/10261) (Figure 1).

The diagram illustrates the number of screened individuals, participants with positive LDCT findings (Lung-RADS 4A/4B/4X), acceptance and refusal of recommended invasive diagnostic procedures, and subsequent diagnostic and treatment steps. Participants may have undergone more than one invasive diagnostic procedure.

### 3.2. Diagnostic Pathway Following Positive LDCT Findings

Of the 479 LDCT-positive individuals, 419 (87.5%) proceeded with recommended invasive diagnostic evaluation, while 60 (12.5%) refused to undergo any invasive diagnostic procedure within six months following a multidisciplinary team (MDT) recommendation.

Refusal of invasive diagnostic procedures was observed across all Lung-RADS subcategories, occurring in 7.9% (8/101) of participants with Lung-RADS 4A findings, 13.3% (16/120) of those with Lung-RADS 4B findings, and 14.0% (36/258) of those with Lung-RADS 4X findings.

Overall, 332 patients (69.3%) underwent at least one invasive diagnostic procedure during follow-up. The distribution of invasive diagnostic procedures and procedure-related safety outcomes is summarized in Table 1.

### 3.3. Procedure-Related Complications and Mortality

Procedure-related complications were uncommon. Among patients undergoing surgical procedures, complications were recorded in 15 cases (6.8%). The complications included: prolonged air leak in 2 cases, cardiac decompensation in 3 patients, and prolonged stay in the ICU in 10 patients. Pneumothorax occurred in 3 patients (9.4%) undergoing transthoracic needle biopsy. There were no procedure related complications related to bronchoscopy. No procedure-related mortality was observed.

### 3.4. Timing of Invasive Diagnostic Procedures

Time intervals between LDCT screening and subsequent invasive diagnostic procedures were calculated for bronchoscopy and surgical interventions. TTNB was not included in time-to-procedure analyses due to the relatively small number of cases and heterogeneity in clinical indications and referral pathways.

All time-to-procedure analyses were restricted to a maximum follow-up of 300 days following LDCT screening. Overall, invasive diagnostic procedures were performed within clinically acceptable time frames, with variability observed across procedure types, screening centers, and screening years.

#### 3.4.1. Time from LDCT to First Bronchoscopy

Among participants with positive LDCT findings who underwent bronchoscopy (*n* = 117), the overall median time from LDCT screening to first bronchoscopy was 36.0 days (interquartile range [IQR] 25.0–47.0), with a mean of 42.9 ± 33.0 days (range 0–224 days; *n* = 117) (Table S1).

**Figure 2:**
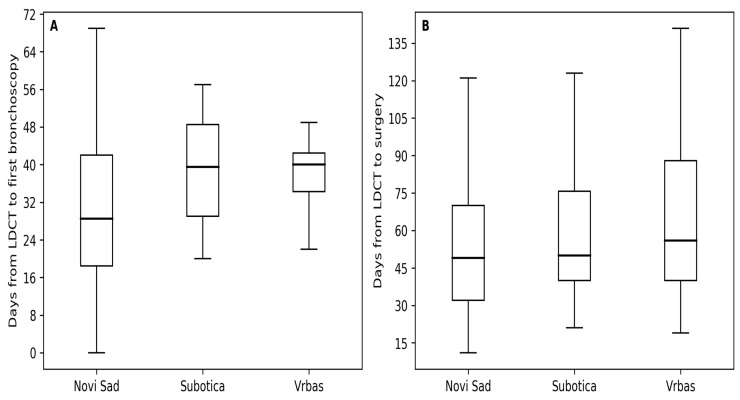
Time from LDCT to first bronchoscopy (**A**) and surgical treatment by screening center (**B**) (Novi Sad, Subotica, Vrbas); (median and interquartile range shown; outliers suppressed for clarity).

When stratified by screening center, statistically significant differences in time to bronchoscopy were observed (Kruskal–Wallis test: *H* = 10.34, *p* = 0.0057). Median time to bronchoscopy was shortest in Center 1 (29.0 days; IQR 20.0–42.0), compared with Center 2 (40.0 days; IQR 29.0–49.0) and Center 3 (41.0 days; IQR 35.0–44.0), although interquartile ranges partially overlapped (Table S2; Figure 2).

Analysis by LDCT screening year also demonstrated significant variation in time to bronchoscopy (Kruskal–Wallis test: *H* = 14.65, *p* = 0.012). Median time increased from 7.0 days in 2020 to values ranging between 35.0 and 40.0 days in subsequent years, with greater variability observed from 2022 onward (Table S3). Despite these fluctuations, all observed time intervals remained within the predefined maximum follow-up period of 300 days.

#### 3.4.2. Time from LDCT to Surgical Treatment

Overall, 218 participants underwent surgical treatment within 300 days following LDCT screening. The median time from LDCT screening to surgery was 52.0 days (IQR 36.5–76.5), with a mean of 63.4 ± 43.7 days (range 7–296 days) (Table S4).

When analyzed by screening center, no statistically significant differences in time to surgery were observed (Kruskal–Wallis test: *H* = 1.04, *p* = 0.594). Median times to surgery were comparable across centers, ranging from 49.0 to 55.0 days, with overlapping interquartile ranges (Table S5; Figure 2).

Similarly, time from LDCT to surgery did not differ significantly across LDCT screening years (Kruskal–Wallis test: H = 9.59, *p* = 0.0877). Although some variability and a gradual increase in median time were observed in later years, these differences did not reach statistical significance within the 0–300 day interval (Tables S6 and S7).

### 3.5. Comparison of Individuals Who Refused Versus Accepted Invasive Diagnostic Procedures

Baseline demographic, clinical, and perceptual characteristics of individuals who refused invasive diagnostic procedures compared with those who accepted diagnostic work-up are summarized in Table 2.

Individuals who refused invasive diagnostics had significantly higher cumulative smoking exposure, a higher prevalence of multimorbidity, and a higher prevalence of previous malignant disease. Refusal rates also differed significantly across screening sites and levels of perceived lung cancer risk. No statistically significant differences were observed between groups with respect to age, sex, body mass index, education level, employment status, living arrangement, or current smoking status.

### 3.6. Multivariable Analysis of Refusal of Invasive Diagnostic Procedures

In multivariable logistic regression analysis, higher smoking exposure was independently associated with refusal of recommended invasive diagnostic procedures (OR per pack-year 1.02, 95% CI 1.00–1.03, *p* = 0.020). Multimorbidity was a strong predictor of refusal (OR 3.45, 95% CI 1.61–7.38, *p* = 0.001), as was a history of previous malignancy (OR 2.92, 95% CI 1.16–7.35, *p* = 0.023). In contrast, greater concern about personal lung cancer risk was associated with a lower likelihood of refusal (OR 0.54, 95% CI 0.29–0.99, *p* = 0.045). Compared with Novi Sad, participants screened in Subotica had a significantly higher likelihood of refusing invasive diagnostics (OR 2.40, 95% CI 1.21–4.78, *p* = 0.012), whereas no significant difference was observed for Vrbas (Table 3).

The final model included 461 participants and demonstrated good calibration (Hosmer–Lemeshow *p*= 0.88) and acceptable discrimination (AUC 0.73, 95% CI 0.66–0.80).

## 4. Discussion

In this real-world multicenter LDCT lung cancer screening program, a meaningful proportion of individuals with positive screening results declined recommended invasive diagnostic procedures. Importantly, refusal was observed among participants who had already engaged with screening and received a clear multidisciplinary recommendation for further diagnostic evaluation. This finding indicates that post-screening attrition cannot be explained solely by barriers to screening access, but rather represents a distinct outcome shaped by a combination of patient-related and system-level factors extending beyond the initial LDCT examination.

Unlike the broad concept of loss to follow-up commonly used in lung cancer screening studies to describe non-adherence across the screening continuum [19], refusal reflects an active decision-making process rather than passive disengagement [12]. By operationally defining refusal as the absence of invasive diagnostic procedures within six months following an MDT recommendation, our study provides a more granular understanding of where post-screening pathways may falter in routine practice. Recognizing refusal as a separate implementation outcome is essential, as it implies different corrective strategies than those aimed at improving attendance or follow-up alone [13].

Consistent with this conceptual distinction, real-world data indicate that completion of recommended follow-up after positive LDCT findings is frequently suboptimal. In a multicenter cohort, adherence within recommended timeframes was 42.6% overall and varied substantially by Lung-RADS category, ranging from 30.0% for Lung-RADS 3 to 49.5% for Lung-RADS 4A and 68.0% for Lung-RADS 4B/4X [9].

Refusal was observed across all Lung-RADS subcategories, occurring in 7.9% (8/101) of participants with Lung-RADS 4A findings, 13.3% (16/120) of those with Lung-RADS 4B findings, and 14.0% (36/258) of those with Lung-RADS 4X findings. Although refusal appeared somewhat more frequent in higher-risk categories, these data suggest that refusal is not confined to a single clinical subgroup and may reflect broader determinants of post-screening decision-making.

Several patient-related clinical characteristics were independently associated with refusal of invasive diagnostics. Multimorbidity emerged as the strongest independent predictor, underscoring the role of cumulative health burden in shaping post-screening decision-making. Individuals with multiple chronic conditions may perceive invasive diagnostic procedures as disproportionately risky or burdensome, particularly in the context of competing health priorities and reduced physiological reserve [9]. A history of prior malignancy was also independently associated with refusal, potentially reflecting accumulated treatment burden and heightened anticipatory distress related to further diagnostic procedures. Cancer survivors may experience fatigue, anxiety, and reduced tolerance for additional medical interventions, particularly when faced with renewed diagnostic uncertainty, which may influence their willingness to undergo invasive diagnostics in the post-screening setting [20].

Notably, while individual comorbid conditions were not significant when examined separately, the composite measure of multimorbidity showed a strong association with refusal. This finding supports the use of aggregated comorbidity measures in screening research and suggests that cumulative health burden, rather than single diagnoses, may be more relevant to decision-making in complex care pathways [12]. From an implementation perspective, multimorbidity may reflect not only biological vulnerability, but also increased healthcare workload, including frequent medical visits, multiple diagnostic procedures, and polypharmacy. In such contexts, the additional burden of invasive diagnostic evaluation following screening may be perceived as disproportionate, particularly within health systems characterized by fragmented care pathways and high organizational demands.

Smoking-related variables warrant particular attention in this context. Although current smoking status was not independently associated with refusal, cumulative smoking exposure (pack-years) remained a significant predictor in multivariable analysis. This finding suggests that long-term smoking history, rather than current behavior alone, may shape perceptions of vulnerability, fatalism, or anticipated benefit from further diagnostic evaluation [12]. Beyond its role as a marker of cumulative biological risk, long-term smoking exposure may also influence psychological responses to screening outcomes. Qualitative evidence suggests that smoking-related stigma, self-blame, and fear of judgment can shape how individuals interpret lung cancer risk information and engage with recommended diagnostic pathways. Such factors may contribute to anticipatory distress and reluctance to undergo invasive diagnostic procedures, particularly in the context of repeated medical encounters following a positive screening result [21].

Perceived concern about personal lung cancer risk was associated with refusal of invasive diagnostics in univariable analyses and remained a borderline but statistically significant predictor after adjustment for clinical and organizational factors (OR 0.54, 95% CI 0.29–0.99; *p* = 0.045). This inverse association suggests that individuals who perceive themselves to be at higher risk may be more likely to engage with recommended post-screening diagnostic evaluation, potentially reflecting greater acceptance of the need for diagnostic clarification. Importantly, the modest magnitude of this effect indicates that risk perception does not operate in isolation but is shaped by—and may interact with—accumulated health experiences (e.g., multimorbidity and prior malignancy) and the broader context of the post-screening pathway.

Evidence from LDCT screening cohorts further supports the link between smoking burden and perceived risk: cumulative smoking exposure (pack-years) and current smoking have been associated with higher perceived personal lung cancer risk, and perceived risk aligns more closely with modeled risk than purely affective responses such as “worry” [22]. This distinction is relevant for interpreting our findings, as a deliberative appraisal of risk may promote diagnostic engagement, whereas affective responses—potentially shaped by stigma, self-blame, fatalism, or anticipatory distress—may influence downstream decisions through different mechanisms [15]. Rather than diminishing the relevance of risk perception, our results underscore its contextual and potentially modifiable role in the post-screening phase.

In this context, risk perception should be viewed as a dynamic construct that may evolves throughout the screening and diagnostic pathway, underscoring the role of healthcare professionals at multiple levels—from primary care and nursing staff to radiologists, pulmonologists, and surgeons—in supporting clear, empathetic, and context-sensitive shared decision-making [10,11]. Interventions that improve understanding of recommendations and address patient concerns may therefore reduce post-screening attrition without compromising patient autonomy.

Emerging evidence suggests that patient understanding of screening results and recommendations, perceived personal risk, and the quality of communication during shared decision-making substantially shape downstream diagnostic decisions following positive LDCT findings. Importantly, however, refusal of invasive procedures is not unique to the screening context. In other lung cancer screening programs, refusal of recommended invasive diagnostics has been reported in approximately 0.7% of screened participants, while population-based data indicate that around 3–4% of patients with resectable lung cancer decline surgical treatment [23]. Recent studies further indicate that both patient-facing decision-support tools and clinician-related factors—including training, confidence in shared decision-making, and referral practices—may influence whether recommended diagnostic pathways are pursued, underscoring refusal as a modifiable, system-mediated outcome rather than a purely individual choice [10,11,22].

Although education level, employment status, and living arrangement were not statistically significant predictors of refusal in this cohort, their consideration remains relevant for contextual interpretation [16]. The screened population predominantly consisted of older individuals, many of whom were retired and living with multiple chronic conditions. In such contexts, logistical burden, healthcare fatigue, and reliance on local healthcare infrastructure may influence decision-making, even when not captured as independent predictors in retrospective analyses. These factors underscore the multifactorial nature of refusal and caution against interpreting it as a purely individual choice.

Differences in refusal rates and time to first invasive diagnostic procedures across screening sites likely reflect organizational characteristics inherent to centralized, real-world lung cancer screening programs. Screening site remained independently associated with refusal after multivariable adjustment, suggesting that center-level factors may shape post-screening decision-making beyond individual clinical characteristics [17,18]. Screening site likely functions as a proxy for geographic accessibility, referral coordination, and local implementation practices, including communication pathways between screening and diagnostic services [24]. Notably, longer median times from LDCT to first bronchoscopy were observed in both Subotica and Vrbas compared with Novi Sad. These differences should be interpreted within the healthcare system context, as Novi Sad functions as a tertiary referral center where most invasive diagnostic procedures for the program are performed, including referrals from both Subotica and Vrbas, which are general hospitals with limited on-site invasive diagnostic capacity. While time to bronchoscopy was not identified as an independent predictor of refusal, variability in early diagnostic timelines may contribute to uncertainty and disengagement during the post-screening phase. Importantly, center-level variability was accounted for in multivariable analyses, and the associations between patient-related factors and refusal persisted after adjustment for screening site, supporting the robustness of the observed findings and underscoring early invasive diagnostics as a potential system-level leverage point rather than a source of site-specific performance differences.

From an implementation perspective, refusal of invasive diagnostics may be considered a meaningful indicator of lung cancer screening program performance, particularly in centralized screening models. Rather than reflecting individual non-adherence alone, refusal may signal misalignment between patient expectations, provider communication, and system organization during the transition from screening to diagnostic evaluation [13].

An important observation of this study is the contrast between relatively uniform time to surgical treatment across screening centers and greater variability in time to first bronchoscopy. Comparable timelines have been reported in other real-world lung cancer screening programs. In a large screening cohort, the median time from LDCT to first tissue sampling was 42 days (IQR 27–70), and the median time to surgery was 52 days (IQR 36–75), closely aligning with the timelines observed in our program [25].

Substantial delays in post-screening diagnostic evaluation have also been reported elsewhere. In a multisite screening program, delays occurred in 47% of positive examinations, with a median delay of 104 days, and were more pronounced among lower-suspicion categories [26]. In addition, temporal fluctuations in bronchoscopy timing likely reflect external system pressures, including periods of reduced procedural capacity during the COVID-19 pandemic. Importantly, variability in bronchoscopy timing should not be interpreted as a direct cause of refusal. In this analysis, time-to-bronchoscopy metrics were available only among individuals who underwent bronchoscopy and were therefore not evaluated as predictors of refusal. Rather, these findings highlight early invasive diagnostics as a potential leverage point for system-level optimization.

Once patients entered definitive surgical pathways, timelines appeared largely standardized, suggesting that downstream treatment processes were robust to local organizational differences. In contrast, bronchoscopy represents an earlier and more operationally sensitive diagnostic step, potentially influenced by local infrastructure, availability of trained personnel, and referral logistics.

Ensuring timely, predictable, and clearly communicated access to minimally invasive diagnostic procedures may reduce uncertainty and support informed decision-making following positive screening results [11,13]. Earlier guidance from the American College of Chest Physicians recommended surgery within 4–8 weeks of referral, whereas more recent recommendations emphasize that interventions to improve timeliness should be developed locally by addressing barriers specific to the local setting [27,28].

Taken together, these findings underscore that LDCT lung cancer screening should be conceptualized as a longitudinal process extending beyond image acquisition to encompass post-screening diagnostic decision-making. While patient education and risk communication remain central to improving diagnostic adherence, system-level factors shaping early diagnostic steps warrant equal attention. Strengthening coordination between screening sites and diagnostic services, enhancing patient navigation, and supporting workforce capacity where feasible may help reduce post-screening attrition without compromising patient autonomy or safety [11,13]. Such measures should be viewed as supportive strategies to improve continuity and equity of care, rather than as critiques of existing healthcare structures.

This study has limitations inherent to its retrospective design. Psychological, cognitive, and socioeconomic factors underlying patients’ decisions were not systematically assessed using validated instruments, limiting insight into the subjective mechanisms driving refusal of invasive diagnostics. The number of refusal events constrained more detailed subgroup analyses. Although the events-per-variable ratio was at the lower margin of conventional recommendations, model calibration was adequate, and covariates were selected a priori based on clinical relevance. Future prospective studies should incorporate validated measures of health literacy, decisional conflict, anxiety, and social support, as well as qualitative approaches, to more comprehensively characterize patient decision-making processes and inform targeted interventions.

## 5. Conclusions

Refusal of invasive diagnostic procedures following positive LDCT screening represents a meaningful implementation challenge, reflecting the combined influence of patient-related vulnerability and system-level organization of post-screening care. Rather than indicating individual non-adherence alone, refusal may signal misalignment between patient expectations, clinical communication, and diagnostic pathways. Targeted interventions focused on risk communication, coordination between screening and diagnostic services, and support for patients with complex health profiles may improve diagnostic continuity and enhance the real-world effectiveness of lung cancer screening programs.

## Figures and Tables

**Table 1:** Diagnostic procedures and safety outcomes following positive LDCT screening.

Variable	*n* (%)
Accepted invasive diagnostic work-up	419 (87.5)
Refused invasive diagnostic work-up	60 (12.5)
≥1 bronchoscopy	118 (24.8)
≥1 TTNB	32 (6.7)
≥1 surgical procedure	218 (45.5)
Surgical complications	15 (6.8)
TTNB complications (pneumothorax)	3 (9.4)
Procedure-related mortality	0 (0.0)

The table summarizes acceptance and refusal of invasive diagnostic procedures, distribution of bronchoscopy, transthoracic needle biopsy (TTNB), and surgical interventions, as well as procedure-related complications.

**Table 2:** Characteristics of participants according to refusal of invasive diagnostic procedures.

Variable	Refused Invasive Diagnostics (*n* = 60)	Did not Refuse Invasive Diagnostics (n = 419)	*p*-Value
Sex, *n* (%)			0.417
Male	33 (55.0)	207 (49.4)	
Female	27 (45.0)	212 (50.6)	
Age, years *	66.7 ± 5.2	65.3 ± 6.1	0.064
BMI, kg/m^2^ *	24.5 ± 4.2	25.5 ± 4.6	0.132
Screening center, *n* (%)			0.004
Novi Sad	21 (35.0)	230 (54.9)	
Subotica	30 (50.0)	122 (29.1)	
Vrbas	9 (15.0)	67 (16.0)	
Place of residence, *n* (%)			0.751
Rural	21 (35.0)	137 (32.9)	
Urban	39 (65.0)	279 (67.1)	
Education level, *n* (%)			0.858
Primary school	12 (20.0)	81 (19.3)	
Secondary school	41 (68.3)	278 (66.3)	
College/University	7 (11.7)	60 (14.3)	
Living arrangement, *n* (%)			0.288
Single	4 (6.7)	23 (5.5)	
Divorced	12 (20.0)	49 (11.7)	
Widowed	12 (20.0)	83 (19.8)	
Living with partner	32 (53.3)	264 (63.0)	
Employment status, *n* (%)			0.291
Employed	10 (17.5)	110 (26.3)	
Unemployed	7 (12.3)	58 (13.8)	
Retired	40 (70.2)	251 (59.9)	
Current smoker, *n* (%)			0.364
Yes	54 (90.0)	359 (85.7)	
No	6 (10.0)	60 (14.3)	
Pack-years *	51.0 ± 23.3	42.6 ± 17.8	0.014
Multimorbidity, *n* (%)			<0.001
Yes	17 (28.3)	42 (10.0)	
No	43 (71.7)	377 (90.0)	
Number of comorbidities *	0.68 ± 0.83	0.48 ± 0.69	0.082
Previous malignancy, *n* (%)			<0.001
Yes	12 (20.0)	22 (5.3)	
No	48 (80.0)	397 (94.7)	
Concern about personal lung cancer risk, *n* (%)			0.039
Rarely	32 (53.3)	156 (37.2)	
Occasionally	22 (36.7)	183 (43.7)	
Often	6 (10.0)	80 (19.1)	

* Continuous variables are presented as mean ± standard deviation. Categorical variables are presented as number (%). Comparisons were performed using the χ^2^ test or Fisher’s exact test for categorical variables, and the independent samples *t*-test or Mann–Whitney U test for continuous variables, as appropriate.

**Table 3:** Multivariable logistic regression analysis of factors associated with refusal of recommended invasive diagnostic procedures among LDCT-positive participants.

Variable	OR	95% CI	*p*-Value
Pack-years (per unit increase)	1.017	1.003–1.032	0.020
Multimorbidity (yes vs. no)	3.451	1.614–7.377	0.001
Previous malignancy (yes vs. no)	2.921	1.161–7.350	0.023
Concern about personal lung cancer risk (present vs. rare/absent)	0.537	0.293–0.986	0.045
Screening site			0.037
Subotica vs. Novi Sad	2.404	1.210–4.779	0.012
Vrbas vs. Novi Sad	1.503	0.619–3.646	0.368

Abbreviations: OR, odds ratio; CI, confidence interval. Odds ratios (ORs) > 1 indicate a higher likelihood of refusing invasive diagnostic procedures. The dependent variable was refusal of invasive diagnostics (1 = refused, 0 = did not refuse). The model included 461 participants due to complete-case analysis. Model fit was adequate (Hosmer–Lemeshow test, *p* = 0.88).

## Data Availability

Data supporting reported results can be obtained from the corresponding author.

## Supplementary Materials

The following supporting information can be downloaded at: https://www.mdpi.com/article/10.3390/diagnostics16050709/s1, Table S1. Histopathological diagnoses among patients with positive Lung-RADS findings; Table S2. Time from LDCT to first bronchoscopy—overall (≤300 days); Table S3. Time from LDCT to first bronchoscopy by screening center (≤300 days); Table S4. Time from LDCT to first bronchoscopy by screening year (≤300 days); Table S5. Time from LDCT to surgery—overall (≤300 days); Table S6. Time from LDCT to surgery by screening center (≤300 days); Table S7. Time from LDCT to surgery by screening year (≤300 days).
