# Supplementary material for: Independent Predictors Associated with Patient Refusal of Invasive Diagnostic Procedures After Positive LDCT Lung Cancer Screening
Source: Diagnostics (Basel). 2026 Feb 27;16(5):709. doi: 10.3390/diagnostics16050709 (PMC12984325; doi:10.3390/diagnostics16050709)
Supplement: Supplementary file 1 [file diagnostics-16-00709-s001.zip › diagnostics-4166445-supplementary.pdf]

Supplementary Materials

Time-to-procedure comparisons across screening centers and screening years were performed using the Kruskal–Wallis test.

Table S1. Histopathological diagnoses among patients with positive Lung-RADS findings.

Diagnosis	n
Adenocarcinoma	117
Squamous cell carcinoma	48
Other NSCLC (including neuroendocrine tumors)	20
Carcinoid tumor	7
Small cell lung cancer	11
Other malignant diagnoses	13
Benign findings	76

Table S2. Time from LDCT to first bronchoscopy—overall (≤300 days).

n	Mean ± SD (days)	Median (IQR)	Min–Max
117	42.9 ± 33.0	36.0 (25.0–47.0)	0–224

Table S3. Time from LDCT to first bronchoscopy by screening center (≤300 days).

Screening center	n	Mean ± SD (days)	Median (IQR)	Min–Max
Center 1	53	36.3 ± 33.8	29.0 (20.0–42.0)	0–224
Center 2	47	50.6 ± 35.8	40.0 (29.0–49.0)	20–195
Center 3	17	41.8 ± 13.7	41.0 (35.0–44.0)	22–76

Table S4. Time from LDCT to first bronchoscopy by screening year (≤300 days).

Year	n	Mean ± SD (days)	Median (IQR)	Min–Max
2020	2	7.0 ± 5.7	7.0 (5.0–9.0)	3–11
2021	11	23.1 ± 12.7	27.0 (12.0–30.0)	8–49
2022	9	55.3 ± 65.8	40.0 (23.0–47.0)	10–224
2023	25	43.5 ± 25.6	35.0 (27.0–54.0)	13–113
2024	47	46.3 ± 35.2	40.0 (25.0–52.5)	0–195
2025	23	42.8 ± 20.0	37.0 (32.5–45.0)	21–109

Table S5. Time from LDCT to surgery—overall (≤300 days).

n	Mean ± SD (days)	Median (IQR)	Min–Max
223	63.4 ± 43.7	52.0 (36.5–76.5)	7–296

Table S6. Time from LDCT to surgery by screening center (≤300 days).

Screening center	n	Mean ± SD (days)	Median (IQR)	Min–Max
Center 1	125	61.6 ± 43.1	54.0 (34.0–72.0)	7–294
Center 2	67	64.5 ± 45.3	49.0 (39.0–76.5)	21–296
Center 3	31	68.2 ± 43.5	55.0 (40.5–83.0)	19–217

Table S7. Time from LDCT to surgery by screening year (≤300 days).

Year	n	Mean ± SD (days)	Median (IQR)	Min–Max
2020	11	46.1 ± 51.6	22.0 (16.5–50.5)	7–170
2021	17	56.1 ± 46.1	39.0 (30.0–65.0)	11–174
2022	18	58.5 ± 28.3	47.5 (37.2–83.8)	17–110
2023	42	69.1 ± 40.9	62.5 (41.0–80.8)	21–192
2024	63	70.9 ± 59.0	53.0 (33.5–88.0)	17–296
2025	72	58.9 ± 29.3	52.5 (40.8–69.2)	19–217
